# Associations of Body Mass Index (Maternal BMI) and Gestational Diabetes Mellitus with Neonatal and Maternal Pregnancy Outcomes in a Multicentre European Database (Diabetes and Pregnancy Vitamin D and Lifestyle Intervention for Gestational Diabetes Mellitus Prevention)

**DOI:** 10.5402/2012/424010

**Published:** 2012-06-12

**Authors:** Akke Vellinga, A. Zawiejska, J. Harreiter, B. Buckley, G. Di Cianni, A. Lapolla, R. Corcoy, D. Simmons, J. M. Adelantado, P. Damm, G. Desoye, R. Devlieger, D. Hill, A. Kautzky-Willer, M. Klemetti, E. Mathiesen, P. Rebollo, F. Snoek, M. Tikkanen, D. Timmerman, A. van Assche, M. van Poppel, E. Wender-Oegowska, F. Dunne

**Affiliations:** ^1^School of Medicine, Clinical Science Institute, National University of Ireland, Galway, Ireland; ^2^Akademia Medyczna im Karola Marcinkowskiego, 60-512 Poznan, Poland; ^3^Department of Medicine, Medical University of Vienna, 1090 Vienna, Austria; ^4^Dipartimento di Endocrinologia e Malattie del Metabolismo, Università di Pisa, 56126 Pisa, Italy; ^5^Dipartimento Scienze Mediche e Chirurgiche, Università degli Studi di Padova, 35122 Padova, Italy; ^6^Institut de Recerca de l'Hospital de la Santa Creu i Sant Pau, 08025 Barcelona, Spain; ^7^Institute of Metabolic Science, Addenbrookes Hospital, Cambridge University Hospitals NHS Foundation Trust, Cambridge CB2 0QQ, UK; ^8^Department of Obstetrics, Rigshospitalet, University Hospital of Copenhagen, 2100 Copenhagen, Denmark; ^9^Department of Obstetrics and Gynecology, Medical University of Graz, 8036 Graz, Austria; ^10^University Hospitals Leuven, Katholieke Universiteit Leuven, 3000 Leuven, Belgium; ^11^Recherche en Sante Lawson, 9552 Bronschhofen, Switzerland; ^12^Department of Obstetrics and Gynaecology, Helsinki University Central Hospital, 00029 Helsinki, Finland; ^13^BAP Health Outcomes Research, S.L, 33010 Oviedo, Spain; ^14^Department of Public and Occupational Health, EMGO Institute, VU University Medical Center Amsterdam, 1007 MB Amsterdam, The Netherlands

## Abstract

*Objective*. Assess the impact of Gestational Diabetes Mellitus (GDM) and obesity on neonatal and maternal pregnancy outcomes. *Methods*. Cross-sectional data (3343 pregnancies) from seven European centres were included in a multilevel analysis of the association between GDM/obesity and caesarean section, macrosomia and neonatal morbidities. *Results*. Comparison of databases identified reporting differences between countries due to the inclusion of true population based samples or pregnancies from specialised tertiary centres, resulting in higher prevalences of GDM for some countries. The analysis showed that obesity and GDM were independent risk factors of perinatal complications. Only BMI had a dose-dependent effect on the risk of macrosomia and caesarean section. Both obesity (BMI > 30 kg/m^2^) and GDM were independent risk factors of neonatal morbidities. *Conclusions*. Obesity and GDM were independent risk factors of perinatal complications. The effect of the worldwide obesity and diabetes epidemic is extending to the next generation.

## 1. Introduction

Obesity is a risk factor for the development of gestational diabetes mellitus (GDM) [1], and increased maternal body mass index (BMI) is associated with a greater frequency of complications in pregnancy, at birth and postpartum [2, 3]. An analysis of a combination of retrospective databases from a European multicentre study was performed to assess the prevalence of GDM and obesity in pregnancy. This paper reports an overview of the data as well as an analysis of the independent association of GDM and obesity on caesarean section, macrosomia, and neonatal morbidities.

## 2. Research Design and Methods

Data on pregnancy and birth outcomes were requested from the ten participating countries over a 6-month period between 2008 and 2009. Seven countries provided sufficient data for inclusion, and a minimum dataset was formed including age, pre-pregnancy BMI, GDM, smoking status, PIH, PET, and caesarean section (CS) for the mother and gestational age, birth weight, gender, still birth/neonatal death, and neonatal morbidities for the offspring. Twin pregnancies were excluded. 

Finland submitted full data on GDM pregnancies only, Ireland and Austria had nearly complete data on both maternal BMI and GDM, while the UK, Italy, Spain, and The Netherlands had missing data in maternal BMI or GDM status or both. The UK and Ireland databases were population based, while all other databases were from selective groups. Comparison between women with and without complete data did not show differences.

The presence of PIH or PET or both were combined in “hypertensive disease.” GDM was defined according to the International Association of Diabetes in Pregnancy Study Groups (IADPSG) criteria (75 g oral glucose tolerance test (OGTT) results fasting glucose levels >5.1 mmol/L or 1 hr >10 mmol/L or 2 hr >8.5 mmol/L) and 100 g OGTT test were recalculated [4]. Primary outcomes were CS, macrosomia (birth weight at or above 4 kg), and neonatal morbidities. Neonatal morbidities included hypoglycaemia, jaundice, or respiratory distress syndrome (RDS). The UK did not record hypoglycaemia and only jaundice or RDS was included for neonatal morbidities.

 Variables were compared between women with and without GDM with univariate tests. A multilevel logistic regression analysis (patients within countries) was performed allowing for differences between countries, and adjusted odds ratios and 95% CI were calculated for all risk factors. Data analysis was performed using PASW 18.0 for univariate analysis (SPSS version 18.0) and MLwiN for multilevel analysis [5].

## 3. Results

Four of the centres (Austria, Italy, Spain, and The Netherlands) were specialised tertiary referral centres for high-risk pregnancies, and the UK, Spain, and Italy used a two-step approach for GDM diagnosis with full OGTT data only available for women with a positive challenge test. This resulted in an inflated GDM prevalence. Data reporting varied between countries. Full data were available from seven countries (3343 pregnant women) for which an overview of the percentage of GDM and GDM by maternal BMI category is shown in Table 1. The heterogeneity between countries in maternal BMI is high, and the percentage of obese women ranges from 12.0% in Spain to 41.5% in Finland. The three multilevel models included all variables with a significant association with at least one outcome (Table 2).

Increasing maternal BMI category and maternal age and a lower gestational age were significantly associated with the risk of a caesarean. Increasing maternal BMI and gestational age and the baby's gender being male were significantly associated with macrosomia. GDM was not found to be a significant risk factor for CS or macrosomia. The presence of GDM in the mother was significantly associated with neonatal morbidities but only significantly associated with maternal BMI when categorised into two groups (normal/overweight (<30 kg/m^2^) and obese (≥30 kg/m^2^), odds ratio 1.48 (95% CI 1.01–2.08)). Smoking status was not found to be a significant factor in any of the models.

## 4. Discussion

Both obesity and GDM have an adverse effect on pregnancy outcome even though their relative influence is not always easy to separate [6–9]. Our analysis of European data showed that high maternal BMI and GDM are independent risk factors of perinatal complications. In particular, GDM was independently associated with an increased risk of neonatal morbidities, but only a maternal BMI over 30 compared to under 30 was significant. These results are in line with the analysis performed on Irish data (Atlantic DIP) from a three-year period where a cutoff point for maternal BMI of 28 kg/m^2^ was found to be associated with a significant rise in adverse pregnancy outcomes. For CS and macrosomia, a significant dose-dependent association was identified, increased risk of CS/macrosomia with increasing maternal BMI. This is similar to the findings from a Spanish cohort of 9,270 pregnant women [6]. The relative greater independent influence of increased maternal BMI compared to GDM on the risk of macrosomia and CS confirmed findings from other studies [6, 10, 11].

The data showed differences in practice between countries. The size of the international database allowed the analysis of associations of maternal BMI and GDM with relative rare adverse outcomes, but the inclusion of more high-risk pregnancies due to the type of participating centre resulted in a selective sample and a higher prevalence of GDM in most countries was due to the selected sample and type of participating centre [12]. Italy, The Netherlands, and Spain have data from a high-risk group, which reflects the nature of the centre from which the data were obtained. In contrast, Ireland has a true population-based database, complete for all variables in the presented analysis. The UK and Austria have inflated prevalences; the UK because their population-based data only contained maternal BMI measurements for a selected group of high-risk patients, the Austrian centre, even though population-based, includes more pregnant women from high-risk groups as an academic gynaecology unit. Finland only included full information for women with GDM. The higher prevalence of GDM in most countries is due to the selected sample as well as type of participating centre. Differences in screening practice and policy were discussed in a review of GDM in Europe [13], which also highlights the need for a uniform European approach to GDM. These differences in prevalence of GDM do not influence the association with the outcomes but they affect the generalisability of the findings. However, cautious comparison with other studies is recommended as the presented outcomes were posttreated GDM and not untreated hyperglycemia as in the HAPO study [14].

Clear guidelines on GDM are in place following the HAPO study and new IADPSG criteria for diagnosis [15]. Even though maternal BMI is shown to be an important risk factor of adverse pregnancy outcomes, European guidelines for the management of obesity in pregnancy are not yet available.

## 5. Strengths and Limitations

A major strength of our study is that it includes data from 7 European countries. This strengthens our analysis in that the number of adverse outcomes is sufficient to observe associations. A weakness of the study is that not all countries report all variables. As the prevalence of neonatal morbidities is likely to be higher, our results most likely underestimate the association of neonatal morbidities with maternal BMI and GDM.

A limitation of the analysis was that the database of four countries included relatively more high-risk pregnancies. As a result this dataset is not a representative sample and findings have to be interpreted within this framework. However, the findings do reiterate previous findings from other more representative populations (association of CS and macrosomia with maternal BMI) and confirm new information (association of neonatal morbidities with GDM and obesity). Even though the population is not representative, the risk factors are suggestive that this might be applicable beyond this varied population as the associations remain similar irrespective of the inclusion or exclusion of variables and countries.

Another limitation of the study was the limited data available from some counties on various associated risk factors, such as socioeconomic status, ethnicity, prior CS, parity, and weight gain during pregnancy. To obtain comprehensive and uniform information from all countries, information on all these factors should be collected. Nevertheless, this analysis highlights that maternal BMI shows a strong association with adverse pregnancy and birth outcomes and that GDM is an independent predictor of neonatal morbidities.

## 6. Conclusion

This European database analysis showed GDM to be independent risk factors of neonatal morbidities as well as confirmed that increased maternal BMI increases the risk of caesarean section, macrosomia, and neonatal morbidities. The effect of the worldwide obesity and diabetes epidemics is extending into the health of the next generation.

## Figures and Tables

**Table 1:** Overview of the number, percentage GDM, and percentage of GDM in each maternal BMI category by country. Higher prevalence of GDM reflects a more specialised centre or the inclusion of GDM pregnancies only.

Country	*N*	GDM (%)	In each maternal BMI category (%)			
			<25	25–30	≥30	≥35
Austria	873	27.6	33.4	37.8	19.7	9.0
Finland	172	99.4	27.9	30.8	22.7	18.6
Ireland	1399	11.8	41.0	36.5	14.6	7.9
Italy	248	69.0	64.5	18.5	10.1	6.9
The Netherlands	67	61.2	52.2	29.9	13.4	4.5
Spain	159	56.0	63.5	24.5	8.2	3.8
UK	426	21.1	45.8	25.4	12.9	16.0
Overall	3344	28.9	42.0	33.1	15.5	9.4

**Table 2:** Adjusted odds ratios and 95% confidence interval (CI) for the multilevel analysis with outcome caesarean section (CS), macrosomia, and neonatal morbidities (NM) corrected for other confounding factors, ^∗^indicates significant odds ratios.

	Outcome CS		Outcome macrosomia		Outcome NM	
	OR	95% CI	OR	95% CI	OR	95% CI
Maternal age	1.04^∗^	1.03–1.06	1.00	0.98–1.02	1.01	0.99–1.02
Gestational age	0.94^∗^	0.91–0.96	1.58^∗^	1.50–1.64	0.93^∗^	0.90–0.97
Boy	1.01	0.87–1.17	1.59^∗^	1.28–1.98	1.03	0.80–1.34

Maternal BMI						
<25	Reference		Reference		Reference	
25–30	1.62^∗^	1.34–1.94	1.84^∗^	1.42–2.37	1.06	0.77–1.46
≥30	1.99^∗^	1.60–2.48	2.37^∗^	1.74–3.26	1.25	0.86–1.78
≥35	2.18^∗^	1.66–2.87	3.71^∗^	2.64–5.21	1.32	0.86–1.90
GDM	0.99	0.82–1.19	0.89	0.66–1.18	1.42^∗^	1.03–1.90
Macrosomia	1.14	0.92–1.43			1.44	0.99–2.06
